# Role of Quetiapine in the Prevention of ICU Delirium in Elderly Patients at a High Risk

**DOI:** 10.2478/jccm-2024-0032

**Published:** 2024-10-31

**Authors:** Walid Y. Kamel, Heba Y. Kamel, Ibrahim M. Elsherif

**Affiliations:** Department of Anesthesia, Intensive Care and Pain Management, Ain Shams University, Abbasiya, Cairo, Egypt; Geriatrics and Gerontology department, Faculty of Medicine, Ain Shams University, Abbasiya, Cairo, Egypt

**Keywords:** CAM-ICU, elderly, delirium, ICDSC, quetiapine

## Abstract

**Background:**

The aim of the present study was to denote the effectiveness of Quetiapine as additive to preventive bundle of delirium in elderly patients with multiple risks for delirium.

**Patients and methods:**

The study was performed on 90 elderly patients over 60 years. The patients were divided into Group Q (Quetiapine) and Group C (No Quetiapine). Delirium was assessed using Intensive Care Delirium Screening Checklist (ICDSC) and the Confusion Assessment Method for the ICU (CAM-ICU).

**Results:**

The incidence of delirium was significantly higher in group C. The severity of delirium was higher among group C; however, it was not statistically significant. The dominant type of delirium was hypoactive in group Q whereas hyperactive in group C. The interrater reliability between CAM-ICU-7 and ICDSE showed a kappa 0.98 denoting excellent correlation between the two scores. Somnolence was the most common side effect of Quetiapine (25%) followed by dry mouth (18%).

**Conclusions:**

Prophylactic low dose of Quetiapine in elderly population in the preventive bundle could reduce the incidence of delirium with a low incidence of a major side effect, as well as CAM-ICU-7 is as effective as ICDSC in monitoring and early diagnosis of delirium.

## Introduction

Delirium is a mental state characterized by confusion and a disturbance in the level of consciousness, making it difficult for the person to focus, sustain attention, or remain attentive. The incidence of delirium ranges from 20–50% in non-ventilated patients and 60–80% in mechanically ventilated patients [1, 2]. Delirium increases the risk of mortality, morbidities including hospital stay and prolonged intensive care unit (ICU) [3, 4], functional disability, early dementia, delayed cognitive disorders [5] and increased costs [6].

Elderly is more vulnerable to acute stress than younger patients [7], because of age related diminution of physiological reserves, greater association of chronic disease in older adults. Assessing vulnerability and diminution of certain system could be quantitative e.g. serum creatinine in kidney function while others could be challenging as in brain vulnerability, Despite of the clinical frailty scale to assess the liability of geriatric population to adverse outcomes [8].

Moreover, hospitalization make delirium more common in elderly patients due to disturbance of patient’s daily home activities to a hospital room. Confusional states can be deteriorated by sensory impairment as well as unintended adverse consequences from interventions [9].

Multiple risk factors have role in the development of delirium [10, 11]. some of those are modifiable. These risk factors have an additive effect including but not limited to mechanical ventilation, high APACHE II score, multiorgan failure, polytrauma, certain medications such as opioids analgesics, pain, catheterization, age, hypertension, interruption in sleep patterns, and smoking [12, 13].

Reducing incidence and duration of delirium and consequently the associated adverse outcome could be not only through the activation of delirium and preventive strategies by correcting modifiable risk factors [14, 15]. But also, through adoption of a bundle of care by using evidence based interventions [13].

Delirium rooms have been designed in some hospital to deliver a specialized care for disoriented patients using the TADA approach (Tolerate, Anticipate, and Don’t Agitate) [16].

The American Geriatrics Society and the American College of Surgeons recommended in guidelines of the prevention and treatment of postoperative delirium, against the use physical or pharmacologic restraints (e.g., antipsychotics, benzodiazepines), since restraints, may increase the risk of falling in this population, unless there is concern for impending risk to the patient or caregivers [17, 18].

Delirium can be classified into two groups according to behavioral activity of psychomotor, that is hypoactive and hyperactive types. Hypoactive delirium is state of decreased consciousness, indifference, withdrawal and is often not diagnosed due to its vague manifestations. In contrast, hyperactive delirium is characterized by restlessness, emotional instability and a moving agitated state that can be easily recognized. Both categories are associated with negative clinical outcome and increased mortality rates [19].

The prevalence and consequences of the delirium, made the society of critical care medicine recommend the necessity of screening of the ICU patients with a screening tools [20], as Intensive Care Delirium Screening Checklist (ICDSC) [1] and the Confusion Assessment Method for the ICU (CAM-ICU) [21].

Quetiapine is atypical antipsychotic which stabilizes mood and reduce agitation by dopamine and serotonin antagonism. It could prevent delirium by its sedation features with lower extrapyramidal side effects.

The aim is to denote the effectiveness of Quetiapine as additive to preventive bundle of delirium in elderly patients with multiple risks for delirium.

## Patients and methods

### Study population

The study was conducted on 90 elderly patients at Geriatric ICU in Ain Shams University hospitals starting from 1^st^ of February 2023 till 1^st^ of October 2023.

### Ethical approval and clinical trial registration

This study was carried out after approval of the research ethics committee of the faculty of medicine, Ain Shams University with approval number (FMASU R292/2022/2023) and conducted in accordance with the principles of the declaration of Helsinki. Informed consent was obtained from each participant. This study is a double blinded clinical trial registered on clinical-trials.gov with a registration number (NCT05793632).

### Sample size and study groups

The Sample size was calculated using Power Analysis and Sample Size (PASS) 15 program, setting power at 80 %, α error at 0.05, on assumption that the incidence of delirium in control group would be 50% and the Quetiapine will result in an absolute risk reduction of 25%. It estimated that sample size of ≥44 patients per group will be needed to detect the difference between the 2 groups regarding incidence of delirium.

The patients were divided into 2 equal groups randomly, Group Q and Group C (45 patients per group). Randomization was done by number lists generated by computer and concealment by the use of sequentially numbered opaque sealed envelopes.

### Inclusion Criteria

The patients with multiple risks for delirium including but not limited to anemia, sedated patients, hypotensive patients, immobilized patients and patients with visual or auditory impairment were included in the study

### Exclusion Criteria

Patients diagnosed with delirium or contraindication for Quetiapine intake were excluded from the study. Also, patients or guardians who refused to participate were excluded from the study.

### Study outcomes

The primary outcome of the study was to study if Quetiapine could help in preventing elderly patients with multiple risks from developing delirium.

The secondary outcome of the study was to compare the Confusion Assessment Method for the ICU (CAMICU-7) versus ICDSC (Table 1) for diagnosis of delirium in the elderly patients.

**Table 1.** Intensive care delirium screening checklist (ICDSC)

**Main item**	**Subitem**
1. Altered level of consciousness:	A: No response
	B: Response to intense and repeated stimulation
	C: Response to mild or moderate stimulation
	D: Normal wakefulness
	E: Exaggerated response to normal stimulation
2. Inattentiveness:	Difficulty following instructions or easily distracted
3. Disorientation To time, place, or person	
4. Hallucination-delusion-psychosis:	Clinical manifestation or suggestive behavior
5. Psychomotor agitation or retardation:	Agitation requiring use of drugs or restraints, or slowing
6. Inappropriate speech or mood:	Related to events or situation, or incoherent speech
7. Sleep/wake cycle disturbance:	Sleeping < 4hrs day, walking all the night, sleeping all day
8. Symptom fluctuation Symptoms above occurring intermittently	

### Study Procedures

Upon admission to the ICU, general examination was done, and the cause of admission was documented, the risks for delirium was screened including anaemia, visual or auditory impairment, sepsis or infection, pain, urinary catheterization, metabolic abnormalities, and immobilized patients. Those patients with risks were included in the study. Delirium was excluded for the population of the study using CAM-ICU-7 and ICDSC.

The CAM-ICU-7 has a 7-point rating scale (0–7 points) that is derived from the CAM-ICU and Richmond Agitation and Sedation Scale (RASS) assessments. The CAM-ICU-7 evaluates *change in mental status*, acute change or fluctuating course (score of 0 or 1); *lack of attention* (score ranges from 0–2); *altered level of consciousness if RASS is other than alert and calm ‘zero’* (0 for absent, 1 for altered level [RASS 1 and −1], 2 for severe altered level [RASS >1,<−1]) and *disorganized thinking* (score ranges from 0–2). A patient is estimated to have no delirium if score is 0 −2, mild to moderate delirium if score is 3–5 and severe delirium if score is 6–7 [22].

And the screening for the delirium was done daily, if the score meets the diagnosis of the delirium, the type of the delirium was defined. Quick sequential organ failure assessment score (qSOFA) was done daily.

Laboratory investigations including complete blood count, coagulation profiles and random blood sugar were done daily. Serum creatinine, sodium, potassium, liver enzymes, serum albumin and lipid profile were collected every 3 days. Other laboratory investigations were collected according to clinical situations. The patients were monitored using 5 leads ECG, pulse oximeter and non-invasive blood pressure monitoring.

In Group C: The patients were monitored after activation of the delirium ABCDEF preventive bundles without adding Quetiapine. In Group Q: The patients were monitored after activation of the delirium ABCDEF preventive bundles with low dose of Quetiapine 25 mg/day (AstraZeneca, United Kingdom). The ABCDEF bundles included: element A “regular pain assessment”; element B “both spontaneous awakening and breathing trials”; element C “regular sedation assessment”; element D “regular delirium assessment”; element E “early mobility and exercise” and element F “family engagement and empowerment”.

The side effects for Quetiapine were monitored including hyperglycemia, hyperlipidemia, dry mouth, extrapyramidal manifestations and somnolence. If any developed it was reported.

### Statistical Analysis

All statistical procedures were carried out using Microsoft Excel 365. The median and interquartile range were used for skewed numerical data while percentage and proportions for categoric values. Mann Whitney and student t Tests were used to compare non-parametric and parametric continuous variables between the two study groups respectively. Chi square and Fisher’s exact tests were used to examine the relationship between categorical variables. P-value < 0.05 was considered statistically significant.

## Results

A total of 90 elderly patients (above 60 years) were included in the study, 45 patients in each group. Age was significantly higher in Group C (P=0.02) but there was no statistical significance among the two groups regarding the gender and co-morbidities (P= 0.38 and P=0.5 respectively) (Table 2).

**Table 2.** Demographics and clinical parameters of patients among the two groups

	**Group Q**	**Group C**	**P value**
Age [mean ± SD]	68.7 ± 7.6	71.2 ± 5.7	0.02

Gender [n (%)]			
Male	19 (42%)	15 (33%)	0.38
Female	26 (58%)	30 (67%)	

Co-morbidities [n (%)]			
Respiratory diseases	4 (9%)	5 (11%)	0.5
Metabolic diseases	2 (5%)	8 (18%)	
Cardiac diseases	16 (36%)	17 (37%)	
Neurological diseases	2 (4%)	0 (0%)	
Multiple	20 (44%)	15 (34%)	

qSOFA Score [median (IQR)]	3 (1–6)	3 (1–4)	0.0001

Length of hospital stay [median (IQR)]	15 (10–19)	10 (5–14)	0.001

SD: standard deviation; qSOFA: Quick sequential organ failure assessment

The qSOFA score was higher in group Q with median (IQR) of 3 (1–6) in comparison to group C with median (IQR) of 3 (1–4). The length of the hospital stay were higher in group Q than group C with a median and IQR of 15 (10–19) versus 10 (5–14) in group C (Table 2). Apart from immobilization (P=0.0004), infection (P=0.0001) and visual impairment (P=0.0008), all other comparable risks of ICU delirium showed no statistical significance among the studied groups (Table 3).

**Table 3.** Risk factors for ICU delirium among the two groups

	**Group Q**	**Group C**	**P value**
Immobilization [n (%)]	37 (82%)	21 (47%)	0.0004
Urinary catheter [n (%)]	33 (73%)	26 (58%)	0.12
Infection [n (%)]	37 (82%)	19 (42%)	0.0001
Visual Impairment [n (%)]	8 (18%)	23 (51%)	0.0008
Auditory impairment [n (%)]	11 (24%)	17 (38%)	0.17
Hemoglobin [gm/dl, mean ± SD]	9.7 ± 1.8	9.6 ± 1.5	0.13
Analgesic & Sedative [n (%)]	17 (38%)	16 (36%)	0.83
Shocked [n (%)]	8 (18%)	9 (20%)	0.78
Intubated [n (%)]	2 (4%)	0 (0%)	0.25
Metabolic abnormalities [n (%)]	40 (89%)	34 (76%)	0.09

SD: standard deviation

There was a significant difference between the two groups regarding the incidence of delirium as shown in table 3. In those who developed delirium, the severity of delirium were higher among group C as revealed by higher score for CAM-ICU-7, however it was not statistically significant, where the median and IQR for CAM-ICU-7 were 2 (3) versus 2 (6) for group Q and group C respectively (P=0.37) and ICDSE score 0 (2) versus 0 (4) among group Q and group C respectively (P=0.06) as shown in table 4. The type of delirium as well were different with the hypoactive type being the dominant form in group Q whereas the hyperactive form was the commonest form in group C (Table 5). The interrater reliability between CAM-ICU-7 and ICDSE showed a kappa 0.98 denoting excellent correlation between the two scores.

**Table 4.** CAM-ICU-7 and ICDSE scores among the two groups

	**Group Q**			**Group C**			**P value**

	**Min**	**Max**	**Median (IQR)**	**Min**	**Max**	**Median (IQR)**	
CAM-ICU-7	0	7	2 (0–3)	0	7	2 (0–6)	0.37
ICDSC	0	7	0 (0–2)	0	7	0 (0–4)	0.06

CAM-ICU-7: Confusion Assessment Method for the ICU; ICDSC: Intensive Care Delirium Screening Checklist

**Table 5:** Comparison between the cases presented with delirium among the two groups

		**Group Q** **N (%)**	**Group C** **N (%)**	**P value**
**Delirium**	**No**	34 (75%)	23 (51%)	0.01
	**Hypoactive**	9 (20%)	5 (11%)	
	**Hyperactive**	2 (5%)	17(38%)	

**Fig. 1.**
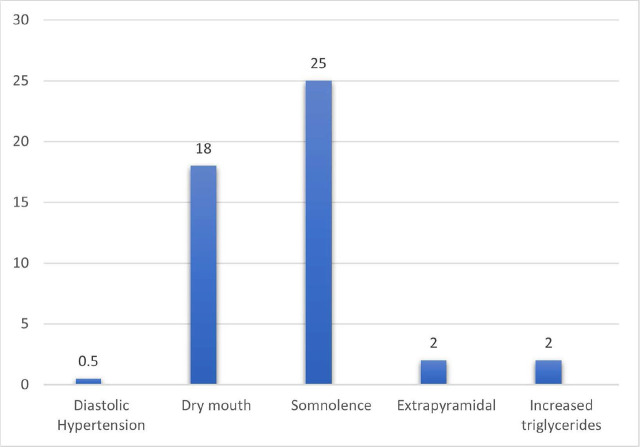
The side effects of Quetiapine among the patients in group Q

Somnolence was the most common side effect of Quetiapine (25%) followed by dry mouth (18%) as shown in figure 1.

## Discussion

Prevention of delirium is a paradigm, a lot of studies have been proposed to decrease or even prevent the poor outcome associated with it, various pharmacological agent and techniques were proposed.

Studies showed that delirium could be prevented in patients undergoing surgery by prevention of pain. In one study ketamine used during cardiac surgeries of 58 elderly patients was associated with a lower rate of postoperative delirium (3%) [23]. But Ketamine still cannot be considered for general usage in prevention of postoperative delirium as further studies are needed with larger sample size, avoid incorporation of nonpharmacologic delirium preventative strategies, and recording long-term outcomes [24].

Not only testing the introduction or use of a pharmacological agent was proposed but the avoidance of certain classes of pharmacological agent was studied to contribute in prevention of delirium.

For example, certain opioids especially pethidine should be avoided in elderly and persons who are susceptible to delirium, as in multiple prospective studies it was associated with an increased risk for delirium [25, 26]. Accordingly, it was recommended for those patients with chronic pain to switch to long-acting opioids such as methadone [27]. Unfortunately, the use of protocols to manage pain result in reducing the severity and duration of delirium, but not its incidence [28] or other postoperative mishaps [23,24,25,26].

Special concerns was directed for elderly population with or without dementia as well as in post stroke setting, where multiple attempts were done to prevent delirium. The potential benefit from using cholinesterase inhibitors (e.g., rivastigmine and donepezil) have been suggested [29, 30], antipsychotic agents and others were all investigated.

However, clinical trials failed to prove decrease incidence and prevalence of delirium in those patients received these medications, moreover the side effects were greater [30].

Low dose of antipsychotic drugs in critical care setting had been studied for prevention of delirium with good results regarding its incidence, severity and duration [31]. However, in one study, it showed increased severity and longer duration of delirium [32].

In the current study, A low dose of Quetiapine decrease the delirium significantly where 11 patients developed delirium versus 22 patients in group C, although there was no a statistically significance difference in most of the risk factors considered in this study and a higher qSOFA in group Q with a median of 3 (1–6) versus 3 (1–4).

The severity of delirium were higher among group C as revealed by higher score for CAM-ICU-7, however it wasn’t statistically significant. Moreover, the dominant type of delirium was the hypoactive type in group Q (9 patients in group Q versus 5 patients in group C) whereas the hyperactive form is the commonest form in group C (2 patients in group Q versus 17 patients in group C). The difference in the age among the two groups may contribute to the difference in the incidence of delirium and the use of quetiapine affects the type of delirium where the hypoactive form was the dominant.

A limited side effect from using Quetiapine was recorded mainly somnolence (25%) followed by dry mouth (18%).

A systematic review and meta-analysis showed that such treatment decreased the incidence of delirium only without reduction of its severity, duration or adverse events [33].It comes to the result that, second generation antipsychotics were found to be more effective than haloperidol. In one study, postoperative low prophylactic dose of dexmedetomidine (0.1 mcg/kg per hour) lowered the incidence of delirium (9% versus 23%; OR 0.35, 95% CI 0.22–0.54). Similar results were supported from different studies [34], side effects of dexmedetomidine were dose dependent bradycardia and hypotension [35].However, these studies weren’t minded with these age group.

The longer length of the hospital stay was higher in group Q than in group C, may be due to higher infection, immobilization and qSOFA in group Q.

The interrater reliability between CAM-ICU-7 and ICDSC showing a kappa 0.98 denoting excellent correlation between the two score in elderly patients. These results match a study by *Krewulak et al.* [36] where it concluded that the agreement between both for delirium measurement was moderate (kappa = 0.51) and fair for measurement of less than clinical threshold symptoms of delirium (kappa = 0.21), however the current study didn’t include subclinical symptoms.

This study has limitations as it didn’t measure the poor outcome associated with delirium, and the significant difference in age group among the two patients’ population may affect the outcome.

In conclusion, prophylactic low dose of Quetiapine in elderly population in the preventive bundle could reduce the incidence of delirium with a low incidence of a major side effect, as well as CAM-ICU-7 is as effective as ICDSC in monitoring and early diagnosis of delirium.
